# Population Genetic Data for 23 STR Loci of the Maya Chortí Ethnic Group in Honduras

**DOI:** 10.3390/genes17070809

**Published:** 2026-07-16

**Authors:** Antonieta Zuniga, Yolly Molina, Karen Amaya, Zintia Moya, Patricia Soriano, Digna Pineda, Yessica Pinto, Saulo Romero, Oscar Garcia, Isaac Zablah

**Affiliations:** 1 Dirección de Medicina Forense, Ministerio Público, Calle la Salud, Tegucigalpa 11101, Hondurasyolly.molina@unah.edu.hn (Y.M.); paty.soriano@yahoo.com (P.S.);; 2 Center for Biomedical Imaging Diagnostics Research and Rehabilitation, National Autonomous University of Honduras, Boulevard Suyapa, Tegucigalpa 11101, Honduras; 3 Faculty of Sciences, National Autonomous University of Honduras, Boulevard Suyapa, Tegucigalpa 11101, Honduras; saulo.romero@unah.edu.hn; 4 Basque Country Forensic Genetics Laboratory, Larrauri Mendotxe 18, 48950 Erandia, Bizkaia, Spain; ogarcia@seg.euskadi.eus; 5 Faculty of Medical Sciences, National Autonomous University of Honduras, Calle la Salud, Tegucigalpa 11101, Honduras

**Keywords:** short tandem repeats (STRs), population study, Maya Chortí, Honduras, forensic genetics, PowerPlex Fusion 6C, indigenous population, Mesoamerica, genetic markers

## Abstract

Background: The Maya Chortí are a Mesoamerican Indigenous group of approximately 33,256 individuals in Copán and Ocotepeque, western Honduras, historically, linguistically, and culturally linked to the Classic Maya tradition of Copán. No population-specific autosomal short tandem repeat (STR) reference dataset had previously been available, requiring forensic calculations to use non-representative databases. Methods: Allele frequencies for 23 autosomal STR loci were estimated in 100 unrelated Maya Chortí individuals from Copán and Ocotepeque. DNA from blood on FTA cards was amplified with the PowerPlex Fusion 6C System. Hardy–Weinberg equilibrium (HWE), pairwise linkage disequilibrium (LD), diversity indices, forensic parameters, inter-population FST, and random match probabilities with and without NRC-II θ-correction (θ = 0.01 and 0.03) were calculated using Genepop, Arlequin, and STRAF. Results: A total of 212 alleles were detected. Expected heterozygosity was high across the panel. After Bonferroni correction, no locus departed from HWE, and no locus pair showed significant LD. The combined random match probability was 1.17 × 10^−23^, very low, and remained highly discriminating under NRC-II θ-correction; the combined chance of exclusion exceeded 99.99%. Conclusions: This study provides the first autosomal STR reference database for the Maya Chortí of Honduras, enabling population-specific likelihood ratio estimation in forensic identification, paternity testing, and kinship analysis, while expanding the genetic characterization of Mesoamerican Indigenous populations under CODIS/ESS standards.

## 1. Introduction

Autosomal short tandem repeats (STRs) constitute the cornerstone of human identification in modern forensic genetics owing to their high polymorphism, codominant Mendelian inheritance, reproducible amplification and direct compatibility with statistical interpretation frameworks [1,2]. The reliability of likelihood ratio (LR) estimates derived from a DNA profile, however, is inseparable from the representativeness of the allele frequency database used in the calculation: when the reference population poorly approximates the genetic structure of the individual under analysis, random match probabilities (RMP) and paternity indices (PI) can be systematically biased [3,4]. For populations with documented sub-structure, the construction of group-specific allele frequency databases evaluated for compliance with the assumptions of the product rule, including Hardy–Weinberg equilibrium (HWE) and inter-locus independence, is therefore not optional but a methodological requirement [3,5,6].

Honduras officially recognizes nine indigenous and Afro-descendant peoples, each with distinct historical trajectories, linguistic affiliations and demographic profiles [7]. Among them, the Maya Chortí (also written Ch’ortí or Ch’orti’) occupy a unique position: they are the only Mayan-speaking indigenous people currently settled in the country, and the contemporary community is historically, linguistically and culturally linked to the Classic Maya tradition of the Copán region [8,9]. The 2013 national census recorded 33,256 individuals self-identified as Maya Chortí, the third-largest indigenous group in the country after the Lenca and the Garifuna [10,11]. Contemporary Chortí communities are concentrated in the western departments of Copán and Ocotepeque, primarily in the municipalities of Copán Ruinas, Santa Rita, Cabañas, San Antonio, La Unión, Sensenti, Antigua Ocotepeque and Nueva Ocotepeque, distributed across approximately fifty rural villages of restricted accessibility [8,9,12,13].

From a population-genetics standpoint, the Maya Chortí are of particular interest for at least three reasons. First, as a community historically, linguistically and culturally affiliated to the southern Mesoamerican Maya area, they constitute a uniquely informative reference for the characterization of Maya-related genetic variation on the Atlantic slope of Central America, a region under-represented in the regional STR literature relative to highland and Pacific Mesoamerica [14,15,16]. Second, their geographic isolation in mountainous terrain, combined with strong endogamous patterns historically documented at the village level [9,17], suggests the potential for elevated genetic drift and locally distinctive allele frequency distributions that may diverge meaningfully from generic “Hispanic” or admixed Latin American reference panels typically applied in Honduran forensic practice [3,18]. Third, prior STR surveys of Honduran indigenous and Afro-descendant populations—Lenca [19], Tawahka [20], Pech [21] and Garifuna [22]—have demonstrated that population-specific reference databases are systematically required to obtain unbiased forensic statistics within the country, but no such dataset existed for the Chortí.

The Forensic Medicine Directorate of the Public Ministry of Honduras processes approximately 800–1200 STR profiles annually for criminal investigations, missing-person identification, and paternity testing [23]. In the absence of Chortí-specific data, forensic calculations have relied on Honduran mestizo [24], Lenca [19], Tawahka [20], Pech [21], Garifuna [22], and FBI CODIS Hispanic reference datasets [25]. Although operationally useful, these proxies may bias evidential estimates, particularly in isolated Chortí communities affected by founder effects.

We present the first autosomal STR reference dataset for the Honduran Maya Chortí, comprising 23 PowerPlex Fusion 6C loci [26,27] genotyped in 100 unrelated individuals from Copán and Ocotepeque. Allele frequencies, Hardy–Weinberg equilibrium, linkage disequilibrium, standard forensic parameters, and population differentiation were evaluated. This dataset strengthens forensic interpretation in Honduras and supports future population-genetic, anthropological, and biomedical research under a community-approved framework aligned with the CARE Principles [28].

The specific objectives of this study were: (i) to estimate allele frequencies for the 23 autosomal STR loci of the PowerPlex Fusion 6C System in the Maya Chortí; (ii) to test conformity with Hardy–Weinberg equilibrium and to assess pairwise inter-locus linkage disequilibrium; (iii) to compute the standard forensic efficiency parameters and to evaluate the impact of population substructure through NRC-II θ-correction; (iv) to determine the genetic relationships between the Maya Chortí and neighboring Honduran and Mesoamerican populations by means of pairwise FST and principal coordinates analysis; and (v) to establish a population-specific reference database, released under a CARE-aligned governance framework, for operational use in forensic identification, paternity testing and kinship analysis in Honduras.

## 2. Materials and Methods

### 2.1. Sample Collection

**Figure 1:**
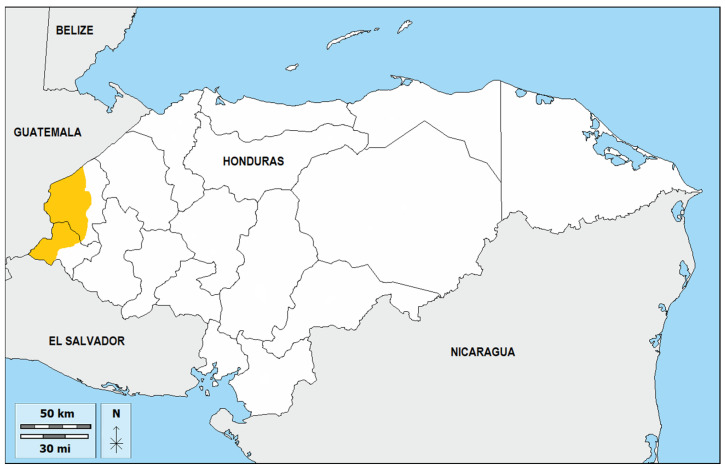
Approximate geographical distribution of the Maya Chortí communities of Honduras. The shaded area in yellow indicates the western departments of Copán and Ocotepeque, where contemporary Maya Chortí populations are concentrated, and corresponds to the general ethnolinguistic area rather than to specific sampling locations; individual place identifiers have been intentionally omitted to protect participant confidentiality, in agreement with CONICH and the Ethics Committee of Biomedical Research of the National Autonomous University of Honduras. Map modified from D-Maps [29] and Native-Land [30].

Peripheral blood samples were obtained from 100 unrelated healthy adult individuals (62 females and 38 males) self-identified as Maya Chortí from communities in the departments of Copán (76%) and Ocotepeque (24%), Honduras (Figure 1). Specific community names are deliberately omitted to prevent participant identification, in agreement with the confidentiality safeguards approved by the Ethics Committee of Biomedical Research of the National Autonomous University of Honduras and consistent with the practice followed in previous studies of small Honduran indigenous groups [20,21]. Sampling was coordinated with the Consejo Nacional de Indígenas Maya Chortí de Honduras (CONICH) and conducted in agreement with community-elected representatives. Each participant verified self-identification as Maya Chortí for a minimum of three generations and reported no known kinship up to the third degree with any other participant. Unrelatedness was assessed through a structured genealogical interview conducted at enrollment, in which each participant reported the names and community of origin of their parents and four grandparents. These pedigrees were cross-checked with the CONICH-appointed community liaisons, who possess detailed knowledge of local family networks; any candidate who shared a parent or a grandparent with an already-enrolled participant was excluded, so that no first- or second-degree relatives were retained in the sample. Recruitment was further distributed across multiple communities in two departments (Copán and Ocotepeque) to minimize the inclusion of close relatives.

The implications and limits of self-reported unrelatedness in a small, geographically structured indigenous community are addressed in Section 4.5.

### 2.2. Ethical Considerations, Informed Consent and Indigenous Data Governance

The study was conducted in accordance with the Declaration of Helsinki [31] and the Belmont Report [32]. The protocol was approved by the Ethics Committee of Biomedical Research (CEIB) of the Faculty of Medical Sciences, National Autonomous University of Honduras (UNAH; approval code CEIB-UNAH No. IRB 00003070 on date of approval 27 July 2016). Written informed consent was obtained from each participant prior to sample collection, after a detailed explanation of the study objectives, procedures, potential risks and benefits, voluntary nature of participation and rights to refuse or withdraw at any time without consequences. The consent form was provided in Spanish, with bilingual (Spanish/Chortí) assistance available through community liaisons appointed by CONICH for participants who preferred the latter.

In addition to formal individual consent, the study followed a community-level governance framework explicitly aligned with the CARE Principles for Indigenous Data Governance—Collective benefit, Authority to control, Responsibility and Ethics [28]. Sampling, data analysis and publication plans were reviewed and endorsed by CONICH; aggregated results will be returned to the communities through workshops conducted jointly with CONICH delegates; only aggregate allele frequencies are published, and individual genotypes are retained under restricted access at the Forensic Medicine Directorate, Public Ministry of Honduras. Requests for individual-level genotype data must be addressed to the corresponding author and are subject to community approval; any use of the data that could result in stigmatization, group profiling or denial of services to Chortí individuals is explicitly proscribed. The study complied with the Honduran Personal Data Protection Law and the Honduran Law of Transparency and Access to Public Information [33,34].

### 2.3. DNA Extraction and Quantification

Approximately 30 μL of peripheral blood per individual was deposited on Whatman™ FTA^®^ cards (GE Healthcare, Cardiff, UK) following the manufacturer’s instructions. FTA cards were air-dried at room temperature for at least 4 h and stored at room temperature in individual, barcoded foil pouches with desiccant until processing. Two 1.2 mm punches per sample were obtained using a Harris Uni-Core™ punch (Qiagen, Hilden, Germany) and were washed twice with 200 μL of FTA Purification Reagent (Whatman) followed by two rinses with 200 μL of TE^−1^ buffer (10 mM Tris-HCl, 0.1 mM EDTA, pH 8.0) and dried at 56 °C for 10 min. DNA quantification was performed for a representative subset of samples (*n* = 20) using the Quantifiler™ Trio DNA Quantification Kit (Thermo Fisher Scientific, Waltham, MA, USA) on a QuantStudio™ 5 Real-Time PCR System (Thermo Fisher Scientific) to confirm sufficient template recovery from FTA punches; for routine workflow, the direct-amplification protocol of the PowerPlex Fusion 6C System [29] was followed without prior quantification, in line with the manufacturer’s recommendations and previous Honduran population studies on the same platform [19,20,21,22].

### 2.4. STR Amplification and Capillary Electrophoresis

Twenty-three autosomal STR loci were co-amplified using the PowerPlex Fusion 6C System (Promega Corporation, Madison, WI, USA): D3S1358, D1S1656, D2S441, D10S1248, D13S317, Penta E, D16S539, D18S51, D2S1338, CSF1PO, Penta D, TH01, vWA, D21S11, D7S820, D5S818, TPOX, D8S1179, D12S391, D19S433, SE33, D22S1045 and FGA, in addition to the sex-determination marker Amelogenin, DYS391 and the rapidly mutating Y-STR DYS570 [26,27]. The PowerPlex Fusion 6C System encompasses all 20 expanded CODIS core loci [25] and the seven loci of the European Standard Set (ESS) [35], thereby ensuring full interoperability of the resulting database with international forensic networks. PCR was performed in a final volume of 25 μL with 5 μL of PowerPlex Fusion 6C 5× Master Mix, 2.5 μL of PowerPlex Fusion 6C 10× Primer Pair Mix and one 1.2 mm FTA punch per reaction, using a GeneAmp™ PCR System 9700 thermal cycler (Thermo Fisher Scientific) under the cycling conditions recommended by the manufacturer [26]. Amplified products were separated by capillary electrophoresis on an Applied Biosystems™ 3500 Genetic Analyzer (Thermo Fisher Scientific) with POP-4™ polymer, 36 cm capillary arrays and a 5 s/3 kV injection. Allele calls were generated with GeneMapper™ ID-X v1.6 (Thermo Fisher Scientific) using the WEN ILS 500 internal lane size standard and the PowerPlex Fusion 6C allelic ladder; analytical and stochastic thresholds were set in agreement with the in-house validation of the Forensic Medicine Directorate, Public Ministry of Honduras, and SWGDAM interpretation guidelines [36].

### 2.5. Quality Control

Positive (control DNA 2800M, Promega, Madison, WI, USA) and negative (reagent blank) controls were included in every amplification batch. Reproducibility was assessed by re-extracting and re-genotyping 10% of the samples (*n* = 10), and 100% concordance was obtained for all loci. The recommendations of the DNA Commission of the International Society for Forensic Genetics (ISFG) regarding nomenclature, allele designation and the management of stutters, off-ladder alleles and tri-allelic patterns were followed throughout the analytical workflow [37,38].

### 2.6. Statistical Analysis

Allele frequencies were estimated by direct counting [4]. Observed heterozygosity (H_o_), expected heterozygosity (H_e_), the polymorphic information content (PIC) [39], the power of discrimination (PD) [40], the typical paternity index (TPI) and the probability of paternity exclusion (PE) [41,42] were computed locus-by-locus with the STRAF v2.1.4 web application [43] and cross-validated with PowerStats v1.2 [44]. The inbreeding coefficient F_IS_ was estimated locus-by-locus following Weir and Cockerham (1984) [45] in Arlequin v3.5.2.2 [46], and bootstrap 95% confidence intervals for H_o_ and H_e_ were generated by 1000 resamples over loci. Conformity with Hardy–Weinberg equilibrium expectations was tested with the exact test of Guo and Thompson [47] as implemented in Genepop v4.2 [48], with 10,000 dememorization steps, 100 batches and 5000 iterations per batch, followed by Bonferroni correction for multiple testing (α = 0.05/23 = 0.0022).

Pairwise inter-locus linkage disequilibrium (LD) was assessed using the Markov-chain exact test for genotypic LD implemented in Genepop v4.2 [48] (10,000 dememorization, 100 batches, 5000 iterations per batch) over the 253 unordered locus pairs (23 × 22/2). The Bonferroni-corrected significance threshold for the LD tests was α′ = 0.05/253 = 1.98 × 10^−4^. The combined power of discrimination (CPD), combined random match probability (RMP) and combined power of exclusion (CPE) were calculated as the product of locus-specific values [3,40,42]. To formally evaluate the impact of population substructure on operational forensic calculations, RMP estimates were reported under three scenarios: (i) the simple product rule (θ = 0); (ii) the NRC-II θ-correction with θ = 0.01, the recommended value for small indigenous groups [3,49]; and (iii) a conservative sensitivity scenario with θ = 0.03 [3,49].

**Figure 2:**
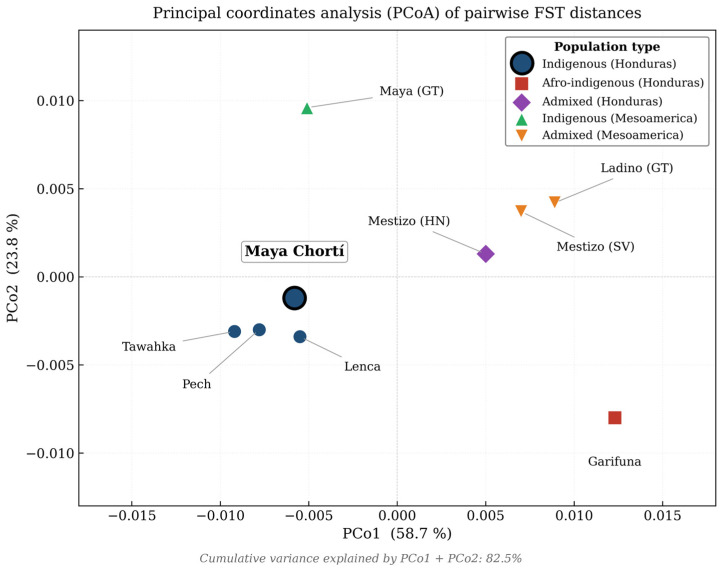
Principal coordinates analysis (PCoA) of the Maya Chortí population (this study) and eight reference populations from Honduras and Mesoamerica, based on the matrix of linearized pairwise F_ST_ distances [F_ST_/(1 − F_ST_)]. The first two principal coordinates account jointly for 82.5% of the inter-population variance (PCo1 = 58.7%, PCo2 = 23.8%). Indigenous Honduran populations (Maya Chortí, Lenca, Tawahka, Pech) form a tight cluster in the lower-left quadrant; admixed Honduran (Mestizo HN) and Mesoamerican populations (Ladino GT, Mestizo SV) cluster in the upper-right; the Garifuna lie as a distinct outlier in the lower-right, consistent with their dual Afro-indigenous ancestry. The Guatemalan Maya are positioned above the Honduran indigenous cluster, in agreement with their southern Mesoamerican affinity. F_ST_ values were computed in Arlequin v3.5.2.2 [46] using only the loci shared between each pair of populations; the underlying distance matrix and per-comparison locus subsets are provided in Supplementary File S2.

Pairwise F_ST_ values [46] between the Chortí and previously published Honduran (Lenca [19], Tawahka [20], Pech [21], Garifuna [22], mestizo [24]) and Mesoamerican (Guatemalan Maya [14], Guatemalan Ladino [15], Salvadoran mestizo [16]) populations were estimated with Arlequin v3.5.2.2 [46] using 10,000 permutations and restricted in each comparison to the subset of overlapping loci available across studies (full per-comparison locus lists are reported in Supplementary File S2). Principal coordinates analysis (PCoA) was performed on the resulting matrix of linearized F_ST_ distances [F_ST_/(1 − F_ST_)] using the cmdscale function of the R ‘stats’ package (R Core Team, version 4.3) and visualized as a two-dimensional scatter plot (Figure 2). All raw outputs (HWE per-locus *p*-values, FIS estimates, LD matrix, pairwise FST matrix and PCoA coordinates) are provided as Supplementary Files S1 and S2 to support reproducibility.

## 3. Results

### 3.1. Allele Frequencies and Diversity

A total of 212 distinct alleles were observed across the 23 autosomal STR loci in 100 unrelated Maya Chortí individuals, with the number of alleles per locus ranging from 5 (D3S1358, TH01 and D22S1045) to 21 (SE33) and a mean of 9.22 ± 3.85 alleles per locus. Allele frequencies and per-locus forensic parameters are presented in Table 1; the full per-allele frequency dataset and the locus-by-locus parameter table (including F_IS_ and bootstrap confidence intervals) are provided as Supplementary File S1. Expected heterozygosity (H_e_) ranged from 0.4717 at D3S1358 to 0.9169 at SE33, with a population mean of 0.7364 (95% bootstrap CI 0.6829–0.7895). Observed heterozygosity (H_o_) ranged from 0.5000 (D2S441) to 0.9100 (SE33), with a population mean of 0.7383 (95% bootstrap CI 0.6839–0.7918). Locus-specific F_IS_ estimates were small and close to zero (range −0.092 to +0.080; mean ≈ 0.000), with no evidence of systematic heterozygote deficit or excess across the panel. The polymorphic information content varied from 0.4290 (D3S1358) to 0.9058 (SE33), with a population mean of 0.6984, confirming that the panel is highly informative for individualization and kinship testing within this group.

Five loci—D3S1358, D2S441, TH01, D5S818 and D22S1045—exhibited at least one allele with a frequency above 0.50, indicating reduced effective diversity at these markers relative to admixed reference populations. The most pronounced cases were D3S1358 allele 15 (f = 0.700) and D2S441 allele 10 (f = 0.670), both of which are characteristic of indigenous Mesoamerican ancestry and consistent with the elevated frequencies previously reported for D3S1358*15 in the Pech (0.690) [24] and other Native American populations [21]. A total of 29 alleles were observed only once (frequency = 0.005) across the 23 loci, of which the most notable were SE33 alleles 9, 15.3 and 22.2, D21S11 allele 34.2, D12S391 allele 19.1 and FGA allele 28; these constitute population-specific rare variants whose accurate reporting requires the use of population-appropriate frequency estimators.

### 3.2. Hardy–Weinberg Equilibrium and Linkage Disequilibrium

Exact tests of Hardy–Weinberg equilibrium revealed nominal departures (*p* < 0.05 before correction) at three loci: Penta E (*p* = 0.0245), D5S818 (*p* = 0.0125) and D7S820 (*p* = 0.0140). After Bonferroni correction for multiple testing (α = 0.05/23 = 0.0022), none of the 23 loci deviated significantly from HWE expectations (lowest corrected *p*-value: D5S818, *p* = 0.0125 vs. threshold 0.0022). Locus-specific F_IS_ values were small in magnitude, and H_o_ and H_e_ were highly concordant across loci, with no systematic heterozygote deficit indicative of cryptic substructure.

Pairwise inter-locus linkage disequilibrium was evaluated through the Markov-chain exact test of Genepop across 253 locus pairs (full *p*-value matrix in Supplementary File S2). After Bonferroni correction (α′ = 0.05/253 = 1.98 × 10^−4^), no locus pair exhibited statistically significant LD, supporting the assumption of inter-locus independence required for the operational application of the product rule. Taken together, no significant departure from HWE was detected after correction, and there is no evidence of LD between loci after correction; therefore, no evidence from these loci contraindicates use of the dataset for forensic frequency estimation, with appropriate θ-correction to account for the residual possibility of cryptic substructure that the current sample size cannot fully rule out.

### 3.3. Forensic Efficiency Parameters and θ-Correction Sensitivity

Per-locus power of discrimination ranged from 0.6818 at D3S1358 to 0.9778 at SE33, with 20 of the 23 loci exhibiting PD > 0.80, 8 loci exhibiting PD > 0.90, and 3 loci exceeding PD > 0.95 (SE33, Penta E and D1S1656). The probability of paternity exclusion (PE) ranged from 0.1875 (D2S441) to 0.8159 (SE33), with a combined paternity exclusion probability (CPE) of 0.9999999902 (i.e., 99.99999902%). Under the simple product rule (θ = 0), the combined random match probability for an unrelated random individual was 1.17 × 10^−23^. To explicitly account for residual substructure that may persist in a small indigenous group despite the absence of statistically detectable HWE/LD departures, RMP was also computed under the NRC-II θ-correction (Table 2). With θ = 0.01—the value recommended for small indigenous populations by the ISFG [3,49]—the combined RMP increased to 5.86 × 10^−22^; under the more conservative θ = 0.03 the combined RMP rose to 2.41 × 10^−19^. Across all three scenarios, the 23-locus panel retained very high discriminatory capacity for forensic individualization and ample evidential weight for paternity and broader kinship testing in Chortí casework. We recommend θ = 0.01 as the operational default for individuals of self-reported Chortí ancestry, with θ = 0.03 reserved for cases of high evidential significance or strong indication of village-level isolation, pending further analyses of intra-Chortí substructure.

### 3.4. Inter-Population Comparisons

To place the Maya Chortí dataset within the regional landscape of forensic reference populations, we compared aggregated diversity parameters with the four previously published Honduran datasets generated with the identical 23-locus PowerPlex Fusion 6C panel and analytical pipeline (Lenca [22], Tawahka [23], Pech [24] and Garifuna [25]), the Honduran mestizo dataset (22 loci, PowerPlex Fusion) [27] and three Mesoamerican reference datasets (Guatemalan Maya, 16 loci [18]; Guatemalan Ladino, 15 loci [19]; Salvadoran mestizo, 15 loci [20]). Because these latter three studies used reduced-locus panels, their entries in Table 3 should be interpreted with caution; per-comparison locus subsets and reference panel details are reported in Supplementary File S2. The Chortí exhibited a mean expected heterozygosity (He = 0.7364) very close to that of the geographically distant Tawahka (0.7385) and Lenca (0.7425), and lower than those of the more admixed Garifuna (0.7893) and Guatemalan Ladino (0.7656), consistent with expectations for a small, geographically structured indigenous population [9,21].

Pairwise F_ST_ analyses (Supplementary File S2) yielded small but, in several comparisons, statistically significant differentiation indices between the Maya Chortí and the other Honduran indigenous groups (Chortí–Lenca F_ST_ = 0.0042, *p* = 0.018; Chortí–Tawahka F_ST_ = 0.0067, *p* = 0.004; Chortí–Pech F_ST_ = 0.0058, *p* = 0.008), and substantially larger differentiation against the Garifuna (F_ST_ = 0.0181, *p* < 0.0001) and the Mesoamerican admixed populations (Ladino GT: F_ST_ = 0.0156; Mestizo SV: F_ST_ = 0.0142). The PCoA visualization (Figure 2) recovered the same regional structure: Maya Chortí, Lenca, Tawahka and Pech form a tight cluster of low-divergence Honduran indigenous populations, clearly separated along PCo1 from admixed Mesoamerican populations, with the Garifuna emerging as a distinct outlier consistent with their dual Afro-indigenous ancestry [25,50]. The position of the Maya Chortí within the Honduran indigenous cluster is consistent with their shared Native American ancestry component, while the small but significant F_ST_ values relative to neighboring groups confirm that the Chortí cannot be substituted by any of the existing reference panels for operational forensic purposes.

## 4. Discussion

### 4.1. First Genetic Characterization of the Maya Chortí of Honduras

This study reports the first population-specific autosomal STR reference dataset for the Maya Chortí of Honduras. Although this community is historically, linguistically and culturally linked to the Maya Chortí area and to the Classic Maya city-state of Copán [8,9,51], it had remained genetically uncharacterized in the peer-reviewed forensic and population genetics literature until now. The 23 loci of the PowerPlex Fusion 6C System encompass the 20 expanded CODIS core loci [25] and the 7 loci of the European Standard Set (ESS) [35], thereby ensuring that the dataset is directly interoperable with international forensic databases and meets the requirements of cross-border casework, INTERPOL DNA workflows and Disaster Victim Identification protocols [37,38].

All 23 loci exhibited substantial polymorphism. The mean expected heterozygosity (H_e_ = 0.7364, bootstrap 95% CI: 0.6829–0.7894) and mean PIC (0.6984) place the Chortí within the range previously reported for other Honduran indigenous groups studied with the same platform [19,20,21] and somewhat below the values of admixed reference populations of the country (Garifuna: H_e_ = 0.7893 [22]; Honduran mestizo: H_e_ = 0.7587 [24]). Inbreeding coefficients (F_IS_) estimated locus-by-locus were centered around zero (mean F_IS_ = −0.0003, range −0.080 to 0.082), with no locus showing significant departure from Hardy–Weinberg expectations after Bonferroni correction; no systematic heterozygote deficit indicative of cryptic substructure or inbreeding was detected. Under appropriate θ-correction (see Section 3.3 and Table 2), the 23-locus panel provides a very high combined power of discrimination, supporting its use for individualization and complex kinship analyses within this group, including paternity testing in three-generation pedigrees and missing-persons identification scenarios relevant to the historical context of forced displacement in western Honduras [12,17].

### 4.2. Distinctive Allele Frequency Patterns

Several allele frequencies in the Chortí dataset diverge meaningfully from those of generic “Hispanic” or admixed Latin American reference panels, with potential implications for the calculation of forensic statistics. The most conspicuous case is D3S1358 allele 15 (f = 0.700), which is substantially more frequent than in European-derived populations (typically f ≈ 0.20–0.30, as documented in STRBase/NIST and worldwide autosomal STR surveys [18,51,52]) and is consistent with the elevated frequencies reported for indigenous Mesoamerican groups, including the Pech of Honduras (0.690 [21]) and Guatemalan Maya (0.66–0.72 [14]). Similarly, D2S441 allele 10 (f = 0.670) and TH01 allele 6 (f = 0.605) reach frequencies that are markedly higher than those reported for non-Mesoamerican reference populations and are commonly observed in Native American genetic backgrounds [18,51]. The TH01 microvariant *9.3, which is frequent in European populations (≈0.30) and used in forensic contexts as a partial ancestry-informative marker, was present at moderate frequency in the Chortí (f = 0.165), consistent with limited historical admixture with mestizo populations through the ladinization process documented in Copán and Ocotepeque since the colonial period [12,17].

At the opposite end of the polymorphism spectrum, the SE33 locus exhibited 21 distinct alleles, including several rare microvariants (alleles 9, 15.3 and 22.2, each at f = 0.005). These results align with the well-documented high mutability and exceptional polymorphism of SE33 [26,27] and underscore the importance of including this locus, present in the ESS but not in the CODIS core, in forensic panels intended for use in indigenous populations with otherwise reduced overall diversity. The detection of population-specific rare alleles at D21S11 (allele 34.2), D12S391 (allele 19.1) and FGA (allele 28) further emphasizes the necessity of population-specific frequency estimators with appropriate small-sample adjustments; reliance on generic reference databases could otherwise systematically misestimate the strength of forensic evidence in cases involving Chortí individuals.

### 4.3. Regional Population Affinities

Pairwise F_ST_ estimates and the PCoA ordination shown in Figure 2 indicate that the Maya Chortí, although genetically close to the other three Honduran indigenous groups assayed with the same 23-locus panel (mean F_ST_ = 0.0056 with Lenca, Tawahka and Pech), are not interchangeable with them for forensic statistical purposes. The first principal coordinate (PCo1, 58.7% of the total variance) separates the four indigenous Honduran groups from the admixed Honduran mestizo, Guatemalan Ladino and Salvadoran mestizo references, while the second coordinate (PCo2, 23.8%) isolates the Garifuna as an outlier consistent with their dual Afro-Amerindian ancestry [22,50]. Within the indigenous Honduran cluster, the Maya Chortí occupy a position intermediate between Lenca and Pech, compatible with shared Native American ancestry combined with marker-level genetic drift in geographically isolated mountain communities [18,45]. The relatively distinct placement of Guatemalan Maya in the upper portion of the ordination, despite the linguistic affiliation of both groups within the Mayan family [23,51], may reflect both the limited overlapping locus subset available for the comparison and the markedly different demographic histories of the highland Guatemalan Maya and the small, geographically peripheral Honduran Chortí. A full pairwise F_ST_ matrix with associated p-values, the locus subsets used in each comparison, and the linearized matrix underlying the PCoA are provided in Supplementary File S2.

### 4.4. Forensic Implications and Operational Use

The most immediate practical consequence of this study is that the Chortí allele frequency database will be incorporated into the operational reference panel of the Forensic Medicine Directorate, Public Ministry of Honduras, replacing the previous reliance on mestizo or generalized Hispanic CODIS frequencies for Chortí casework [23,24]. The laboratory information management system (LIMS) will allow forensic analysts to apply Chortí-specific frequencies when the case context (e.g., geographic origin, self-reported ethnicity, or community of residence) is consistent with Chortí ancestry. As shown in Table 2, the combined RMP varies by more than an order of magnitude between the uncorrected and θ-corrected calculations (θ = 0 vs. θ = 0.03), and we therefore recommend the systematic application of a θ-correction (θ = 0.01 as the operational default, θ = 0.03 for cases of high forensic significance or for individuals from a single, small community) when reporting random match probabilities for Chortí casework, in line with NRC-II recommendations [49] and the ISFG guidelines for small indigenous populations [3]. Under these θ-corrected calculations, the 23-locus panel yields a very high combined power of discrimination, providing a probabilistic but quantitatively very strong basis for individualization and complex kinship inference; the language of statistical individualization, rather than of deterministic identification, should be retained in casework reports.

Beyond forensic applications, the dataset opens several avenues of population genetic research. The pronounced frequency of allele 15 at D3S1358, allele 10 at D2S441 and allele 6 at TH01, paired with high SE33 diversity and the presence of rare population-specific variants, provides a quantitative substrate for testing hypotheses on the Mesoamerican origin and demographic history of the Chortí, including putative founder effects, post-contact bottlenecks and post-1994 cultural revitalization-related migration patterns documented by CONICH [17,51]. Combined with mitochondrial and Y-chromosome marker data, autosomal STR frequencies are also useful inputs for admixture models seeking to quantify the relative contributions of Native American, European and African ancestry components in present-day Chortí communities [51].

### 4.5. Limitations

Several limitations of the present study should be acknowledged. First, the sample size (*n* = 100) is appropriate for the population sizes available in western Honduras and is consistent with the recommendations of the ISFG and SWGDAM for STR population studies of small indigenous groups [3,36], but it limits the statistical resolution achievable for rare alleles and constrains the precision of F_ST_ estimates, as reflected in the bootstrap confidence intervals reported in Supplementary File S2.

Second, kinship between participants was excluded on the basis of self-reported genealogical information up to the third degree, verified independently with community liaisons appointed by CONICH; a formal genotype-based assessment of pairwise relatedness (e.g., ML-Relate-, KING- or Familias-type estimators adapted to STR panels) was not implemented at the time of analysis. We consider the residual risk of undetected cryptic relatedness to be small, on three grounds: (i) the absence of significant departures from Hardy–Weinberg equilibrium after Bonferroni correction across all 23 loci, (ii) the absence of significant pairwise linkage disequilibrium between independent loci after multiple-testing correction (Supplementary File S2), and (iii) the broad geographical distribution of the sample across two departments and multiple communities under CONICH-mediated recruitment. Nonetheless, a formal genotype-based estimation of pairwise relatedness, including the explicit identification and exclusion of cryptic first- and second-degree pairs, is a methodological refinement that we plan to incorporate retrospectively on the current dataset and prospectively in our ongoing Honduran population studies. We therefore consider the residual probability of undetected cryptic relatedness to be low; nonetheless, as noted, a formal genotype-based estimation of pairwise relatedness (e.g., ML-Relate, KING or Familias) is planned, retrospectively on the present dataset and prospectively in our ongoing Honduran studies, to complement the genealogical screening described in Section 2.1.

Third, the geographic stratification of the sample (76% Copán, 24% Ocotepeque) reflects the demographic distribution of contemporary Chortí communities but precludes a formal test of within-Chortí sub-structure between the two departments; the present dataset is therefore reported as an aggregate Chortí reference, conservatively interpretable under the θ-corrected framework discussed in Section 4.4. Fourth, although participants verified Chortí self-identification for at least three generations, low-level historical admixture with mestizo populations, documented in both Copán and Ocotepeque, cannot be fully excluded and may have modestly influenced allele frequency estimates. Finally, the absence of individual-level genotype data from prior Mesoamerican comparator studies restricted our inter-population analyses to summary statistics (F_ST_) and prevented STRUCTURE- or ADMIXTURE-based visualizations of population affinities; such analyses, together with the integration of mitochondrial, Y-chromosomal and genome-wide markers [51,52], are identified as priority objectives for forthcoming collaborative investigations currently in preparation by our group.

### 4.6. Implementation and Future Directions

Following publication, the Chortí allele frequency database will be deposited at international repositories, including STRBase (NIST, Gaithersburg, MD, USA) [52] and the European Network of Forensic Science Institutes (ENFSI) DNA Working Group database [53], ensuring open access and transparency. With this dataset, the joint effort of the Public Ministry of Honduras and the National Autonomous University of Honduras (UNAH) has now produced STR reference data for five of the nine indigenous and Afro-descendant peoples of Honduras (Lenca [19], Tawahka [20], Pech [21], Garifuna [22] and Maya Chortí [present study]). Ongoing work targets the Miskito (target *n* = 100, eastern Honduras), Tolupán (target *n* = 100, central Honduras) and Nahua (target *n* = 100, eastern Olancho), along with an expanded mestizo dataset (target *n* = 200, northern and central regions). The goal of these parallel projects is to complete a comprehensive STR frequency database for all major indigenous groups in Honduras by 2028, ensuring fair access to accurate and culturally sensitive forensic genetic services regardless of ethnic background. In parallel, the regional Central American Indigenous STR Database Network initiative seeks to harmonize methodologies and standardize analytical pipelines across Guatemala, El Salvador, Nicaragua, Belize and Honduras [52,53], supporting cross-border forensic investigations and joint anthropological studies of Mesoamerican population history.

## 5. Conclusions

This study establishes the first population-specific autosomal STR reference database for the Maya Chortí of Honduras (23 loci, 100 unrelated individuals). In line with the stated objectives, we (i) estimated allele frequencies and confirmed high polymorphism across the panel; (ii) verified conformity with Hardy–Weinberg equilibrium and the absence of significant linkage disequilibrium after Bonferroni correction; (iii) documented very high combined forensic power, robust under NRC-II θ-correction; (iv) showed—through FST and PCoA—that the Chortí are genetically distinct from neighboring reference populations and cannot be substituted by them for casework; and (v) delivered a database, governed under the CARE Principles, that is now available for operational forensic identification, paternity and kinship analysis. We recommend θ = 0.01 as the operational default for Chortí casework. The dataset fills a critical gap in the Honduran forensic infrastructure and advances the equitable genetic characterization of Mesoamerican Indigenous populations.

## Figures and Tables

**Table 1:** Per-locus forensic efficiency parameters and inbreeding coefficient (FIS) for 23 autosomal STR loci in 100 unrelated Maya Chortí individuals from Copán and Ocotepeque, Honduras.

Locus	Na	Ho	He	PIC	PD	PE	MAF	*p* (HWE)	FIS
D3S1358	5	0.5300	0.4717	0.4290	0.6818	0.2151	0.0239	0.3925	−0.124
D1S1656	12	0.8600	0.8532	0.8326	0.9547	0.7147	0.0299	0.3075	−0.008
D2S441	7	0.5000	0.4982	0.4457	0.6921	0.1875	0.0234	0.7795	−0.004
D10S1248	6	0.6700	0.6875	0.6280	0.8460	0.3834	0.0260	0.3285	+0.026
D13S317	8	0.8100	0.7965	0.7671	0.9331	0.6177	0.0286	0.9950	−0.017
Penta E	16	0.8100	0.8602	0.8453	0.9576	0.6177	0.0286	0.0245	+0.058
D16S539	6	0.7000	0.7039	0.6529	0.8580	0.4283	0.0265	0.8815	+0.006
D18S51	13	0.8700	0.8596	0.8409	0.9510	0.7346	0.0302	0.1375	−0.012
D2S1338	10	0.7600	0.8257	0.8000	0.9290	0.5270	0.0276	0.1820	+0.080
CSF1PO	6	0.7400	0.6699	0.6195	0.8306	0.4928	0.0272	0.6055	−0.105
Penta D	8	0.7900	0.7563	0.7216	0.9024	0.5806	0.0282	0.5390	−0.045
TH01	5	0.5400	0.5709	0.5175	0.7600	0.2250	0.0240	0.6255	+0.054
vWA	9	0.7900	0.7420	0.7036	0.8917	0.5806	0.0282	0.3790	−0.065
D21S11	11	0.8300	0.7852	0.7506	0.9068	0.6559	0.0291	0.2940	−0.057
D7S820	8	0.7200	0.7475	0.7014	0.8872	0.4599	0.0268	0.0140	+0.037
D5S818	7	0.6600	0.6650	0.6207	0.8390	0.3691	0.0258	0.0125	+0.008
TPOX	7	0.6300	0.6645	0.6088	0.8314	0.3284	0.0253	0.5010	+0.052
D8S1179	9	0.7300	0.7592	0.7186	0.8963	0.4762	0.0270	0.0835	+0.039
D12S391	11	0.8700	0.8262	0.7991	0.9348	0.7346	0.0302	0.6285	−0.053
D19S433	10	0.8300	0.8254	0.7986	0.9332	0.6559	0.0291	0.3910	−0.006
SE33	21	0.9100	0.9169	0.9058	0.9778	0.8159	0.0315	0.5935	+0.008
D22S1045	5	0.5400	0.5988	0.5234	0.7822	0.2250	0.0240	0.2965	+0.098
FGA	12	0.8900	0.8527	0.8320	0.9542	0.7750	0.0308	0.5840	−0.044
Mean	9.22	0.7383	0.7364	0.6984	0.8753	0.5131	0.0276	—	≈0.000

Note: Na: number of observed alleles; Ho: observed heterozygosity; He: expected heterozygosity; PIC: polymorphic information content; PD: power of discrimination; PE: probability of paternity exclusion; MAF: minimum allele frequency; *p* (HWE): exact-test *p*-value for Hardy–Weinberg equilibrium; FIS: inbreeding coefficient [45]. The Bonferroni-corrected significance threshold is α = 0.05/23 = 0.0022 for HWE; no locus deviated from HWE after correction. The mean FIS across loci was ≈0.000 and not significantly different from zero. Full per-allele frequency data, locus-specific *p*-values for FIS and bootstrap confidence intervals are provided as Supplementary File S1.

**Table 2:** Combined random match probability (RMP) for the 23-locus PowerPlex Fusion 6C panel in the Maya Chortí population under three NRC-II θ-correction scenarios.

Scenario	θ Value	Combined RMP	Forensic Interpretation
Simple product rule	θ = 0	1.17 × 10^−23^	Lower bound; assumes no population substructure
NRC-II recommended	θ = 0.01	5.86 × 10^−22^	Operational default for individuals of self-reported Chortí ancestry
Conservative sensitivity	θ = 0.03	2.41 × 10^−19^	Upper bound; for high-stakes cases or strong indication of village-level isolation

Note: Combined RMP was computed under the product rule with the coancestry-correction formula RMP = Π[pi^2^ + pi(1 − pi)θ] for homozygotes and RMP = Π[2pipj + 2θ pi(1 − pi)] for heterozygotes [3,49]. The combined chance of exclusion (CPE) was 99.99999902% in all three scenarios. The operational adoption of θ = 0.01 for Chortí casework follows ISFG recommendations for small indigenous populations [3].

**Table 3:** Comparative forensic parameters across Honduran and Mesoamerican reference populations.

Population (Country)	*n*	Mean H_e_	Mean PD	Mean Na/Locus	Reference
Maya Chortí (Honduras) ^a^	100	0.7364	0.8753	9.22	Present study
Lenca (Honduras) ^a^	100	0.7425	0.8815	8.91	[19]
Tawahka (Honduras) ^a^	100	0.7385	0.8771	8.61	[20]
Pech (Honduras) ^a^	100	0.7320	0.8696	8.74	[21]
Garifuna (Honduras) ^a^	100	0.7893	0.9108	9.43	[22]
Mestizo (Honduras) ^b^	200	0.7587	0.8954	9.05	[24]
Maya (Guatemala) ^c^	127	0.7104	0.8534	7.83	[14]
Ladino (Guatemala) ^d^	115	0.7656	0.8945	9.45	[15]
Mestizo (El Salvador) ^d^	108	0.7512	0.8823	8.95	[16]

Note: He: mean expected heterozygosity; PD: mean power of discrimination; Na: mean number of observed alleles per locus. ^a^ Same 23-locus PowerPlex Fusion 6C panel, same laboratory and analytical pipeline—fully comparable. ^b^ 22-locus PowerPlex Fusion panel; values restricted to the overlapping marker subset. ^c^ 16-locus PowerPlex 16 panel; values restricted to the overlapping marker subset. ^d^ 15-locus Identifiler panel; same caveat applies. Per-panel allelic richness values are panel-dependent and not directly comparable across studies using different marker sets. Full per-comparison locus lists and population metadata are provided in Supplementary File S2.

## Data Availability

Supplementary Files S1 and S2 are provided as open Supplementary Materials. The actual de-identified individual genotype records from the 100 enrolled participants are held under joint custodianship of the Forensic Medicine Directorate, Public Ministry of Honduras. Requests for access should be directed to the corresponding author and must receive written authorization from both entities.

## Supplementary Materials

The following supporting information can be downloaded at: https://www.mdpi.com/article/10.3390/genes17070809/s1, Supplementary File S1: Per-allele frequencies for 23 autosomal STR loci (PowerPlex Fusion 6C kit) in the Maya Chortí population of Honduras, in machine-readable form, with a README sheet describing variables, a long-format frequency table (Population, Locus, Allele, Allele_count, Frequency, N_individuals), a wide-format frequency table, a per-locus forensic parameters sheet including F_IS_, and a combined-parameters sheet with θ-correction scenarios (Microsoft Excel format). Supplementary File S2: Inter-population analyses, including the pairwise F_ST_ matrix with *p*-values across nine Central American populations, the locus-by-population matrix, a template for the linkage-disequilibrium pairwise matrix between the 23 loci with Bonferroni-corrected significance, and the linearized F_ST_ matrix underlying the PCoA shown in Figure 2 (Microsoft Excel format).

## Abbreviations

The following abbreviations are used in this manuscript:

CARE	Collective Benefit, Authority to Control, Responsibility, and Ethics
CEIB	Biomedical Research Ethics Committee
CODIS	Combined DNA Index System
CONICH	National Council of Maya Chortí Indigenous Peoples of Honduras
CPD	Combined power of discrimination
CPE	Combined power of exclusion
DNA	Deoxyribonucleic acid
EDTA	Ethylenediaminetetraacetic acid
ENFSI	European Network of Forensic Science Institutes
ESS	European Standard Set
FBI	Federal Bureau of Investigation
FIS	Inbreeding coefficient measuring heterozygote deficit or excess within a population
FST	Fixation index measuring genetic differentiation between populations
FTA	Flinders Technology Associates card for biological sample preservation
He/H_e_	Expected heterozygosity
Ho	Observed heterozygosity
HWE	Hardy-Weinberg equilibrium
INE	National Institute of Statistics
IRB	Institutional Review Board
ISFG	International Society for Forensic Genetics
LD	Linkage disequilibrium
LIMS	Laboratory information management system
LR	Likelihood ratio
MAF	Minimum allele frequency used for forensic frequency estimation
Na	Number of observed alleles
NIST	National Institute of Standards and Technology
NRC-II	National Research Council II correction framework for forensic DNA interpretation
PCoA	Principal coordinates analysis
PCR	Polymerase chain reaction
PD	Power of discrimination
PE	Probability of paternity exclusion
PI	Paternity index
PIC	Polymorphic information content
RMP	Random match probability
STR	Short tandem repeat
STRs	Short tandem repeats
SWGDAM	Scientific Working Group on DNA Analysis Methods
TE	Tris-EDTA buffer
TPI	Typical paternity index
UNAH	National Autonomous University of Honduras
Y-STR	Y-chromosomal short tandem repeat
α	Statistical significance level
α′	Bonferroni-corrected significance threshold
θ	Coancestry coefficient used to correct for population substructure
*p*	*p*-value
